# Evaluating Variability in Extracellular Vesicle Characterization Across Measurement Techniques

**DOI:** 10.1002/jex2.70154

**Published:** 2026-06-01

**Authors:** Premanshu K. Singh, Ali F. Usmani, Debmalya Halder, Jenna Miller, Tharune Kanagasabai, Patricia Sarchet, Kevin Weller, George J. Klarmann, Raphael E. Pollock, Federica Calore, Shaurya Prakash

**Affiliations:** ^1^ Department of Mechanical and Aerospace Engineering The Ohio State University Columbus Ohio USA; ^2^ Comprehensive Cancer Center The Ohio State University Columbus Ohio USA; ^3^ 4DBio^3^ Center for Biotechnology Department of Radiology and Bioengineering Uniformed Services University of the Health Sciences Bethesda Maryland USA; ^4^ The Geneva Foundation Tacoma Washington USA; ^5^ Department of Cancer Biology and Genetics The James Comprehensive Cancer Center The Ohio State University Columbus Ohio USA

**Keywords:** extracellular vesicles, EV‐DNA, fluorescent NTA, liposarcoma conditioned media, nanoflow cytometry, physical characterization, tetraspanins, urinary EVs

## Abstract

Reliable, reproducible, and standardized characterization of extracellular vesicles (EVs) remains a central challenge due to methodological variability across analytical platforms and intrinsic EV heterogeneity within and across biofluids. Here, we evaluated EVs isolated using ultracentrifugation from dedifferentiated liposarcoma‐conditioned media and commercially available healthy donor urine. Physical characterization and surface tetraspanin profiling of isolated EVs were performed using three widely used instruments: NanoFCM, CytoFLEX Nano, and ZetaView Evolution. All platforms consistently detected small EVs; however, absolute size distributions, particle concentrations, and tetraspanin expression levels varied, reflecting differences in optical configuration, detection principles, and fluorescence background sensitivity. Additionally, EV‐associated DNA was quantified using two different methods, revealing further variability in both the DNA yield and quantity. Together, the observations highlight the method‐dependent nature of EV analyses and underscore the importance of carefully considering instrument‐specific metrics for interpreting EV data across biofluids.

## 1 Introduction

Extracellular vesicles (EVs) are nanoscale, lipid bilayer membrane‐bound particles actively secreted by nearly all cell types into biological fluids such as blood, urine, and saliva (Théry et al. 2006) as well as into tissues (Crescitelli et al. 2025). EVs comprise a heterogeneous population, including exosomes (30–200 nm), microvesicles (100–1000 nm), and apoptotic bodies (>1 µm), each differing in biogenesis, molecular cargo, and biological function (Van Niel et al. 2018, Bebelman et al. 2018, Rädler et al. 2023), with size distributions and nomenclature continuing to evolve (Buzas 2023, Welsh et al. 2024, Yáñez‐Mó et al. 2015). EVs also serve as mediators of intercellular communication by transferring their molecular cargo, including proteins, lipids, RNA, and DNA, between cells (Lee et al. 2024) thereby influencing a wide range of physiological and pathological processes such as immune modulation, cancer progression, and tissue repair (Becker et al. 2023, Kalluri and McAndrews 2023, Adamczyk et al. 2023). Recent studies have highlighted the potential of EVs in disease diagnosis (Jiang et al. 2025), prognostic assessment (Kalluri and LeBleu 2020), therapeutic applications (Xu et al. 2025), and vaccine development (Santos and Almeida 2021).

The ‘Minimum Information for Studies of Extracellular Vesicles’ (MISEV) guidelines, most recently updated in 2023 (Welsh et al. 2024), provide guidance for EV characterization, emphasizing the assessment of size, surface markers, and nucleic acid cargo (Welsh et al. 2024). Despite these guidelines, there are no universally accepted standards for the characterization and analysis of EVs. While several commercial instruments are available for EV analyses, each instrument relies on distinct operating principles, making cross‐platform comparison of the same samples often challenging (Hendrix et al. 2023, Lang et al. 2022). Systematic cross‐platform comparisons of EV physical properties, surface‐marker detection, and EV‐DNA quantification (representing only a subset of the total EV‐associated DNA (Tsering et al. 2024)) across distinct biofluids also remains limited. This lack of comprehensive comparative analysis, in turn, hinders the development of robust, standardized methodologies and tools for EVs characterization. These limitations have also been discussed in previous reviews (Yáñez‐Mó et al. 2015, Arab et al. 2021), and the recent MISEV 2023 guidelines (Welsh et al. 2024).

**Figure 1:**
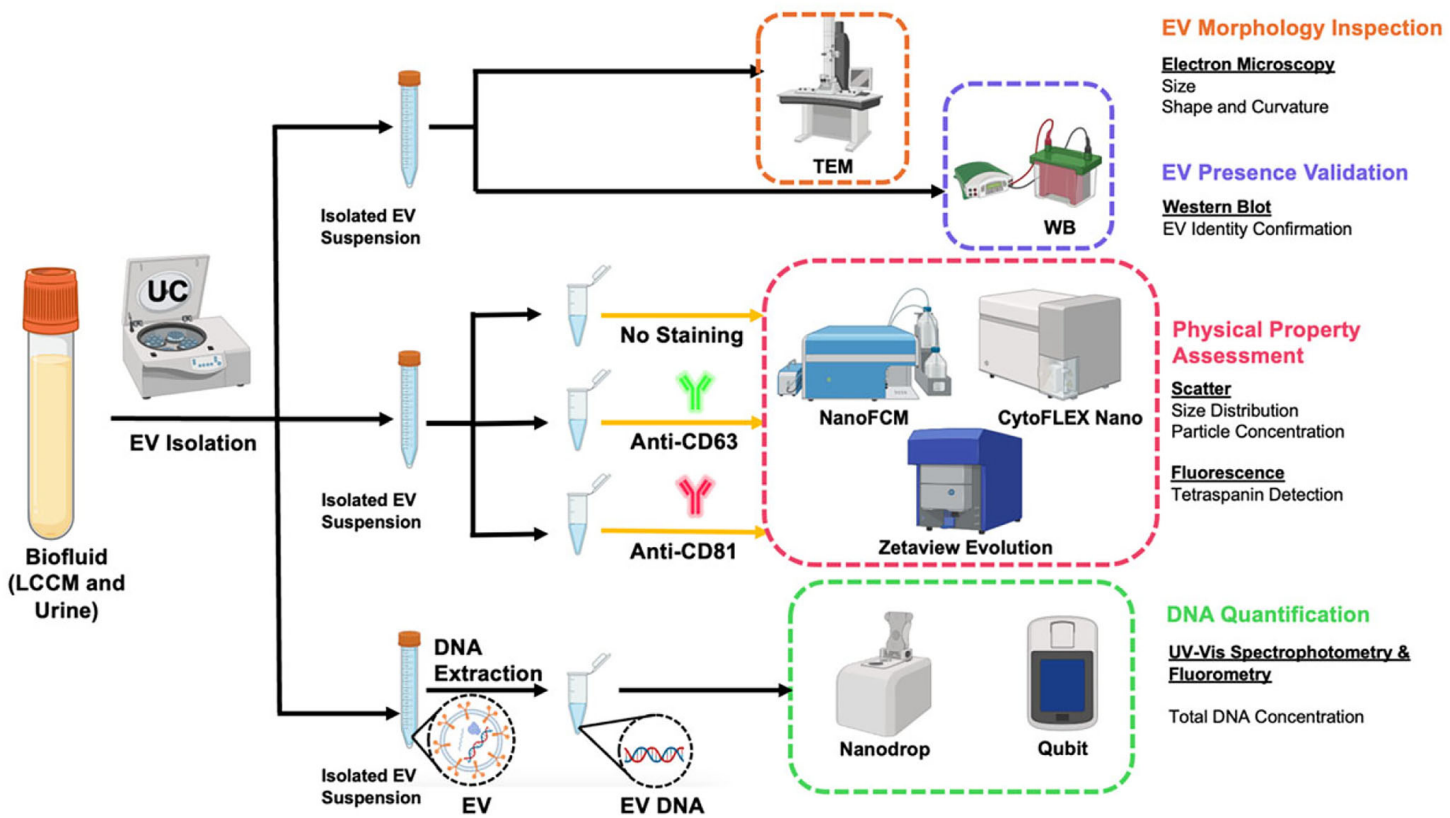
Schematic for the multi‐technique, multi‐parameter characterization of EVs. EVs were isolated from liposarcoma cell‐conditioned medium (LCCM) and healthy donor urine. The isolated EVs were analyzed for size, concentration, presence of surface tetraspanin markers, and DNA cargo in accordance with MISEV 2023 guidelines. EV morphology was visualized by using transmission electron microscopy (TEM), and EV presence was confirmed using Western blot (WB) analysis. For physical EV characterization, three distinct instruments were used: the ParticleMetrix Zetaview Evolution, the NanoFCM, and the CytoFLEX Nano. EV‐DNA was extracted and quantified using spectrophotometry (Nanodrop) and fluorometry (Qubit) (Figure partially assembled using *BioRender*).

In this work, we carried out a comparative assessment of EV physical properties (size and concentration) and the expression of two tetraspanin surface markers (CD81 and CD63) (schematically illustrated in Figure 1) on EVs isolated from liposarcoma‐conditioned culture medium (LCCM) and human, healthy donor urine, following MISEV 2023 guidelines. A multi‐technique characterization approach was employed using three orthogonal analytical platforms: NanoAnalyzer U30 (NanoFCM Inc.), CytoFLEX Nano (Beckman Coulter), and ZetaView Evolution (Particle Metrix). Additionally, transmission electron microscopy (TEM) was used for direct, image‐based quantification of EV size. EV‐associated DNA was extracted using commercial kits (QIAamp DNA kit, Qiagen) (Casadei et al. 2017) and quantified *via* spectrophotometric (NanoDrop; Thermo Scientific) and fluorometric (Qubit; Thermo Scientific) methods (Musante et al. 2020). This multi‐technique, multi‐parameter characterization was performed on EVs derived from a dedifferentiated liposarcoma cell line (Casadei et al. 2022, Casadei et al. 2017), reflecting conditions relevant to past work (Casadei et al. 2017, Musante et al. 2020, Erdbrügger et al. 2021, Casadei et al. 2021), and on EVs isolated from the urine of healthy donors, thereby capturing the complexity of a physiologically relevant biological fluid. The purpose of this study is to provide a systematic evaluation of commonly used EV characterization methods across various instruments and biofluids to highlight method‐dependent differences which in turn may be useful for the future development of standardized analytical strategies.

## 2 Materials and Methods

Isolated EVs were characterized in accordance with MISEV 2023 guidelines to ensure consistency. EV samples were isolated using ultracentrifugation (UC), and their characterization was performed on aliquots from the same EV preparation using multiple analytical platforms to enable direct, cross‐platform comparison. A summary of the specific protocols employed for each analytical technique is provided below.

### 2.1 Cell Culture and Conditioned Media‐Derived EV Preparation

For the isolation of EVs from liposarcoma cell line‐conditioned media (LCCM), Lipo246 cells were cultivated for EV production as previously reported (Casadei et al. 2022, Casadei et al. 2017, Casadei et al. 2021, Peng et al. 2011, Casadei et al. 2019). Briefly, cells were cultured in Dulbecco's Modified Eagle Medium (DMEM; Gibco) supplemented with 10% (v/v) Fetal Bovine Serum (FBS; Corning). Once cells reached approximately 60% confluency, the medium was replaced with serum‐free DMEM (Gibco), and cells were incubated for 48 h to facilitate EV release. The resulting EV‐enriched LCCM was centrifuged at 2000 × g for 20 min at 4°C (5810 R Eppendorf Centrifuge) to remove cell debris, and the clarified supernatant was stored at −80°C until further processing (Casadei et al. 2022). The EVs from LCCM are denoted as LCCM‐EVs.

EVs were isolated by differential centrifugation, following previously published reports (Casadei et al. 2022, Casadei et al. 2017, Casadei et al. 2019, Singh et al. 2025). Briefly, 20 mL of frozen LCCM was thawed at 4°C and centrifuged at 10,000 × g for 30 mins at 4°C using a JA‐10 fixed‐angle aluminum rotor (k‐factor: 3,610) in an Avanti JXN‐30 centrifuge (Beckman Coulter). The resulting supernatant was carefully transferred to ultracentrifugation (UC) tubes (Beckman Coulter, Polycarbonate, 26.3 mL) and spun at 100,000 × g for 70 mins at 4°C using a 50.2 Ti rotor (k‐factor: 157.7) in Optima XPN‐80 ultracentrifuge (Beckman Coulter). EV pellet was washed with 1x phosphate‐buffered saline (PBS) and ultracentrifuged again at 100,000 × g for 70 mins at 4°C to minimize the co‐isolation of contaminants. The final UC pellet was resuspended in 1x PBS and stored at −80°C until further use.

### 2.2 Healthy Donor Urine‐Derived EV Isolation

Commercially obtained healthy donor urine samples (Innovative Research) were processed following previously published protocols (Musante et al. 2020, Barreiro et al. 2020, Sedej et al. 2022). Briefly, raw urine was first centrifuged at 850 × g (5810 R Eppendorf Centrifuge) for 10 min at 4°C to remove cells and large debris. The resulting supernatant was then divided into 20 mL aliquots and stored at −80°C for EV isolation. Separately, a 500 µL aliquot of raw urine was stored for the quantification of urinary creatinine (U‐creatinine), which was subsequently used to normalize urinary analyte levels and account for variations in urine dilution (Erdbrügger et al. 2021). U‐creatinine concentrations were measured using the Creatinine Urinary Detection Kit (Invitrogen, Cat# EIACUN) according to the manufacturer's instructions. In line with published literature, urine‐derived EVs are hereafter referred to as uEVs.

uEVs were also isolated using protocols previously reported (Musante et al. 2020, Gheinani et al. 2018, Hinzman et al. 2022). Briefly, 20 mL of frozen urine was thawed at 4°C. Next, 400 µL of 0.5 M EDTA (pH 8) and 5 mL of 5x PBS were added for inhibition of uromodulin aggregation, Ca‐oxalate formation, and pH neutralization, respectively (Sedej et al. 2022). Samples were then centrifuged at 10,000 × g for 30 mins at 4°C. The supernatant was collected and carefully layered over 4 mL of 30% sucrose cushion in 1x PBS and ultracentrifuged at 100,000 × g for 70 mins at 4°C (Gupta et al. 2018). Following UC, the supernatant was removed, leaving approximately 5 mL of sample above the pellet. The remaining solution was washed with 1x PBS and ultracentrifuged again at 100,000 × g for 70 mins at 4°C. The final supernatant was discarded, and the resulting EV pellet resuspended in 1x PBS and stored at ‐80°C until further analysis. uEV isolation was performed using the same UC rotor setup as for LCCM‐EVs.

### 2.3 Transmission Electron Microscopy Imaging

Morphological assessment of isolated EVs was performed using negative‐staining transmission electron microscopy (TEM) imaging, as reported previously (Singh et al. 2024). Briefly, 7.5 µL of the isolated EV sample was placed on a glow‐discharged formvar‐coated and carbon‐stabilized copper grid (Electron Microscopy Sciences, Hatfield, PA) at room temperature. After 20 min, the TEM grid was washed twice with 1x PBS (pH = 7.4) and then fixed with 7.5 µL of 1.25% glutaraldehyde for 5 mins at room temperature. Post fixation, the TEM grid was rinsed three times with DI water and immediately stained with 1% (w/v) uranyl‐acetate in ethanol for 15 s. The grid was then air‐dried. Imaging was performed using a FEI Tecnai G2 Spirit microscope operated at 80 kV, and micrographs were analyzed in ImageJ (v1.44) to quantify EV size, following the procedure described previously (Singh et al. 2024).

### 2.4 Western Blot

For immunoblotting, EVs were isolated from 40 mL of LCCM and 30 mL of urine. LCCM‐EV and uEV pellets were re‐suspended in 150 µL of 1x PBS. All samples were lysed in ice‐cold 10x RIPA buffer (Invitrogen) supplemented with 100x Protease Inhibitor cocktail (Thermo Scientific) and incubated for 20 min at 4°C. Protein concentration of lysed samples was determined using Pierce BCA Protein Assay Kit (Thermo Scientific). For UC‐isolated EV samples and corresponding supernatants, 4 µg of total proteins were mixed with 4x NuPAGE LDS sample buffer (Invitrogen) and loaded onto a Criterion Tris‐HCl 4–20% precast gel (Bio‐Rad), followed by transfer to a nitrocellulose membrane. For Lipo246 and HepG2 cell lysates, 25 µg of total protein were loaded per lane. Membranes were probed overnight at 4°C with primary antibodies specific to each experiment: Calnexin (Sigma‐Aldrich), CD9 (BioLegend), TSG101 (Sigma‐Aldrich), and Uromodulin (Invitrogen). Incubation with secondary antibodies was performed for 1 h at room temperature. Proteins were visualized by chemiluminescence.

### 2.5 Characterization of Isolated EVs

Isolated EVs were characterized according to their size, concentration, and expression of tetraspanins CD63 and CD81, which are commonly assessed in sarcoma fluid analysis (Gassmann et al. 2021) and urine samples (Yim et al. 2023). All instruments described below were operated according to the manufacturers’ instructions.

For fluorescence‐based surface marker phenotyping, the UC pellet obtained from 20 mL of the biofluid (LCCM or human donor urine) was re‐suspended in 75 µL of particle‐free 1x PBS and divided into three 25 µL aliquots. One aliquot was left unstained to serve as a negative control, while the remaining two aliquots were stained separately with Mouse anti‐human CD81 monoclonal antibody (clone M38, PE‐conjugated; Thermo Scientific, MA1‐10292, RRID AB_11153835) or Mouse anti‐human CD63 monoclonal antibody (clone MEM‐259, Alexa Fluor 488; Thermo Scientific, MA5‐18149, RRID AB_2539523). 0.26 µg of Anti‐CD81‐PE and anti‐CD63‐AF488 antibodies were added to the isolated EV aliquots, and incubated for 30 min at room temperature in the dark (Yim et al. 2023). Unbound antibodies were then removed by UC at 100,000 × g for 70 min at 4°C (Yim et al. 2023). The resulting EV pellets were resuspended in 100 µL of 1x PBS. Antibody‐only process controls were prepared by diluting each antibody in 25 µL of 1x PBS at the same concentrations used for the EV staining, followed by purification using the same UC protocol applied to the EV preparations. These controls were analyzed in parallel with the EV samples to assess non‐specific background signals. The percentage of marker‐positive EVs was calculated as the ratio of fluorescence‐positive events to the total number of particles detected in scatter mode. The scatter‐detected population includes both EVs and non‐vesicular particles.

### 2.6 Nanoparticle Tracking Analysis

Single‐particle analyses in both scatter and fluorescence modes were performed using ZetaView Evolution instrument (Particle Metrix) equipped with a 488 nm laser, following the manufacturer's operating protocol (Particle‐Metrix). EV suspensions were diluted in 1x PBS to a final concentration of 10^7^–10^9^ particles/mL and analyzed using a 500 µL working volume. For the scatter mode, the parameters were set as follows: camera sensitivity at 85%, shutter at 150 µs, frame rate at 50 frames/s, minimal area at 0.5 pixels^2^, maximal area at 500 pixels^2^, minimum brightness at 20 a.u., and minimum trace length at 10 frames. For fluorescence‐mode measurements of stained EVs, a 550 nm long‐pass filter was used, with the following settings: camera sensitivity at 95%, shutter at 150 µs, frame rate at 50 frames/s, minimal area at 0.5 pixels^2^, maximal area at 500 pixels^2^, and brightness at 20–35 a.u. (adjusted for background and target‐to‐particle ratio). ‘Low Bleach’ mode was enabled to minimize photobleaching of the fluorophore.

### 2.7 Nano Flow Cytometry

#### 2.7.1 NanoFCM

The NanoAnalyzer U30 instrument (NanoFCM Inc.), equipped with dual 488/640 nm lasers, was used for the simultaneous detection of side scatter (SSC) and fluorescence (FL) of individual particles, as recommended by the manufacturer's operation manual (NanoFCM). Single‐photon counting avalanche photodiode detectors (SPCM APDs) with bandpass filters allowed for the collection of light in specific channels (SSC‐488/10 nm; FL1‐525/40 nm; FL2‐670/30 nm). The sampling pressure from the air pump module was set to 1 kPa; the sheath fluid was gravity‐fed HPLC‐grade water, and each acquisition was performed over a 60‐s period. For each particle, the peak area was recorded simultaneously in all three detection channels. Samples were diluted to attain a particle count within the optimal range of 2,000–12,000/min (10^7^‐10^9^ particles/mL) (NanoFCM). Blank measurements of particle‐free 1x PBS were acquired and subtracted to calculate final concentration. Particle concentrations and sizing were calibrated by comparison to a standard containing 250 nm fluorescent silica nanoparticles (NanoFCM Inc.) and a proprietary 4‐modal NanoFCM Silica Nanospheres Cocktail #1 (NanoFCM Inc.) (NanoFCM). EV samples measured using the same protocol as calibration beads. FL1‐525/40 nm and FL2‐670/30 nm fluorescent channels were used for the detection of AF488‐conjugated CD63‐stained EVs and PE‐conjugated CD81‐stained EVs, respectively. Gating based on fluorescence intensity enabled the quantification of CD63‐ and CD81‐labelled EV sub‐populations. Data processing was conducted using the NanoFCM NF Profession V3.0 software.

#### 2.7.2 CytoFlex Nano

Flow‐cytometric phenotyping of EVs was also performed using a six‐color, four‐laser CytoFLEX Nano flow cytometer (Beckman Coulter). The instrument was initialized in CytExpert Nano (version 1.2) using the standard CytoFLEX sheath fluid (B51503, Beckman Coulter), and quality control (QC) was carried out according to the manufacturer's protocol (Beckman‐Coulter). For scatter detection, EV suspensions were diluted in 1x PBS to maintain event rates below 3000 events/s (Palviainen et al. 2020, Motori et al. 2020). SSC was collected using the small‐particle detection module on the 488 nm laser, with the violet side‐scatter (VSSC) channel used as the trigger. Size calibration was performed using traceable polystyrene microspheres ranging from 40 to 1000 nm (Thermo Scientific 3000‐Series Nanosphere Size Standards: 3040A, 3080A, 3100A, 3150A, 3200A, 3300A, 3500A, 3700A, and 4010A). The Flow Cytometry Pass (FCMPass) software was used to generate a standardized scatter calibration curve. Fluorescence detection was performed using the factory‐configured optical filters (Beckman‐Coulter) and fluorescence calibration was verified using 500 nm Fluorospheres (C92889, Beckman Coulter). Each experiment included particle‐free 1x PBS blanks and antibody‐only process controls, which were processed identically to the EV samples. Fluorescence‐positive events were quantified after background subtraction, and EV gating was based on combined scatter characteristics and fluorescence signal patterns (Beckman‐Coulter). Data acquisition was performed in CytExpert version 2.6.

### 2.8 EV‐DNA Extraction and Quantification

EV‐DNA extraction and quantification followed previously reported protocols (Casadei et al. 2022, Casadei et al. 2017). For LCCM‐ EVs, the UC pellet derived from 20 mL LCCM was resuspended in 500 µL of 1x PBS. Of this, 400 µL were used for DNA extraction using the QIAamp DNA Micro Kit (Qiagen Inc.) according to the manufacturer's instructions (Qiagen 2014), and the remaining sample was stored at ‐80°C for future analysis. DNA was eluted in 100 µL of deionized (DI) water, concentrated using a Savant SpeedVac DNA130 vacuum concentrator (Thermo Scientific) for 75 mins, and re‐suspended in 5 µL of DI water. EV‐DNA was quantified using two methods: (i) UV absorbance with Nanodrop One C Microvolume UV‐Vis Spectrophotometer (Thermo Scientific) in dsDNA mode and (ii) fluorescence detection with the Qubit dsDNA High Sensitivity Assay Kit (Invitrogen) (Elzanowska et al. 2022), using 1 µL of concentrated DNA per assay. DNA yield was normalized to the biofluid input volume (Welsh et al. 2024).

For uEVs, 220 µL of the UC suspension was used for DNA extraction, while the remaining sample was stored at ‐80°C. Samples were treated with Proteinase K (Invitrogen) to reduce non‐vesicular protein contamination, followed by DNase I (Invitrogen) to remove non‐EV contaminant DNA. To exclude co‐isolation of RNA molecules during DNA purification, samples were also treated with RNase A (Qiagen) (Sedej et al., 2022 Sep 23). Past work has shown that similar treatment conditions preserve EV structural integrity (Sedej et al., 2022 Sep 23, de Voogt et al. 2024, Moon et al. 2019). DNA was extracted using the QIAamp DNA Micro Kit, eluted in 100 µL, concentrated by SpeedVac, and resuspended in 5 µL of DI water. DNA quantification was performed using Qubit fluorometer, with each sample measured in triplicate, and normalized to both urine volume (ng/mL of urine) and urinary creatinine concentration (mmol/L creatinine) (Erdbrügger et al. 2021).

### 2.9 Statistical Analysis

Statistical analyses were performed using JMP 19 (SAS Institute Inc.). Differences in continuous variables between groups were assessed using two‐sided Student's *t*‐tests with a significance threshold of α = 0.05.

## 3 Results

### 3.1 EV Detection

**Figure 2:**
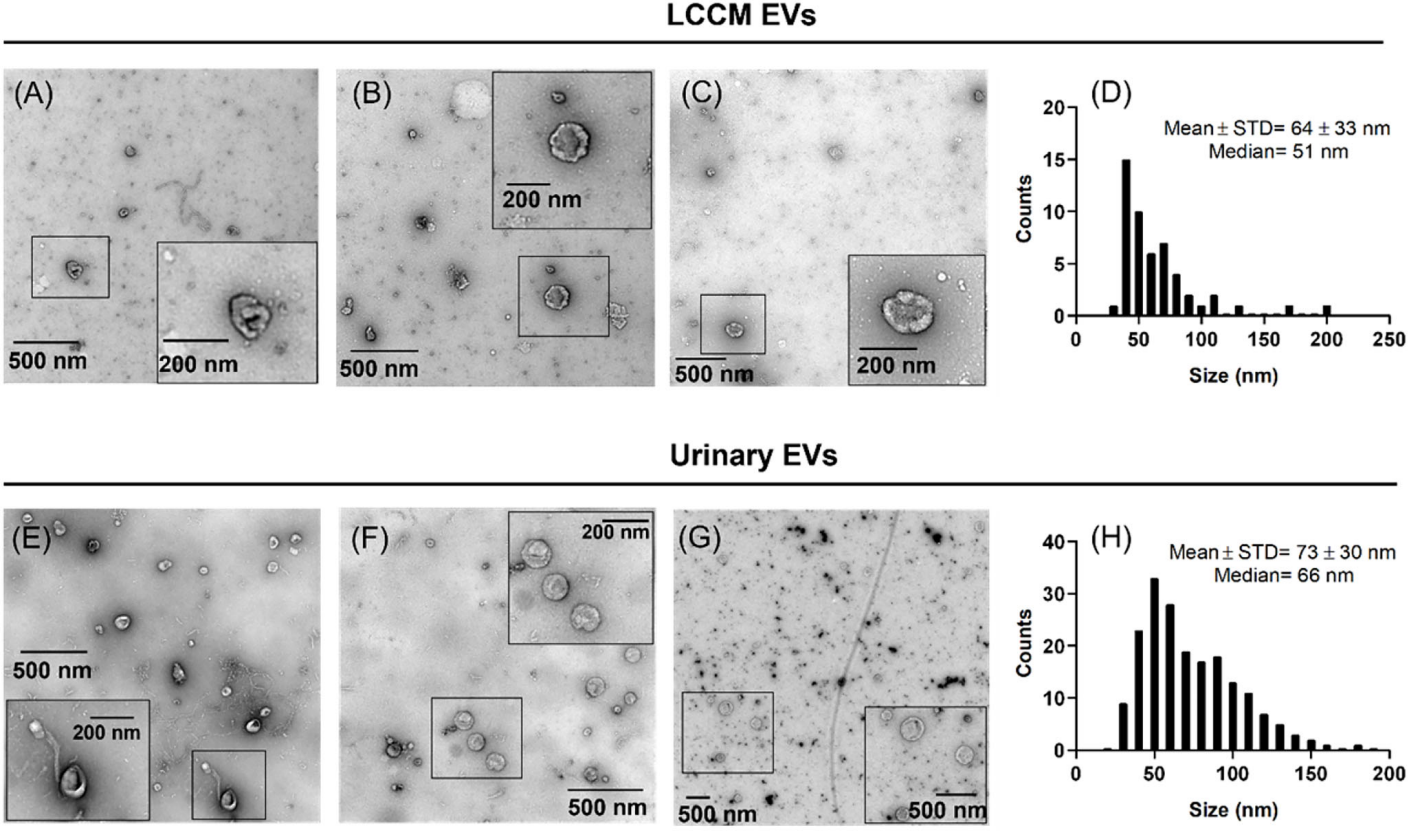
TEM images of isolated EVs. Transmission electron microscopy imaging (A–C) of LCCM‐EVs and (E–G) uEVs shows the characteristic cup‐shaped morphology. Insets highlight representative vesicles at higher magnification, confirming the presence of intact, membrane‐enclosed structures. Histograms (D) and (H) represent particle diameter distributions from TEM image‐based measurements of LCCM‐EVs (64 ± 33 nm (51 images) with a median diameter of 51 nm and uEVs (73 ± 30 nm (190 images) with a median diameter of 66 nm). Each *n* represents a single EV measurement, while noting that an individual image may contain multiple vesicles.

The presence of EVs in both LCCM and urine‐derived preparations was confirmed by TEM and western blot, following MISEV 2023 guidelines (Welsh et al. 2024). TEM imaging revealed distinct, cup‐shaped vesicles with well‐defined bilayer membranes in both sample types (Figure 2), confirming the successful isolation of intact EVs (Singh et al. 2024). LCCM‐EVs exhibited a mean diameter of 64 ± 33 nm and a median of 51 nm (Figure 2D), while uEVs yielded a mean diameter of 73 ± 30 nm with a median of 66 nm (Figure 2H), also consistent with past reports (Singh et al. 2024, Rikkert et al. 2019) for similar EVs.

To evaluate the observed size differences, we performed a non‐parametric Wilcoxon rank‐sum test (as normality assumptions were not satisfied based on a goodness‐of‐fit test), which confirmed that the distributions are statistically significantly different. This difference in mean and median diameters between uEVs and LCCM‐EVs likely reflects differences in biological origin and sample composition (Shukla et al. 2023, Arya et al. 2024). EVs originate from multiple cell types along the nephron and urinary tract and are therefore expected to exhibit greater heterogeneity, including contributions from larger vesicle subpopulations (Erdbrügger et al. 2021). In contrast, LCCM‐derived EVs arise from a more homogeneous cell population, which may result in comparatively smaller and more uniform vesicle sizes (Casadei et al. 2022, Casadei et al. 2021).

**Figure 3:**
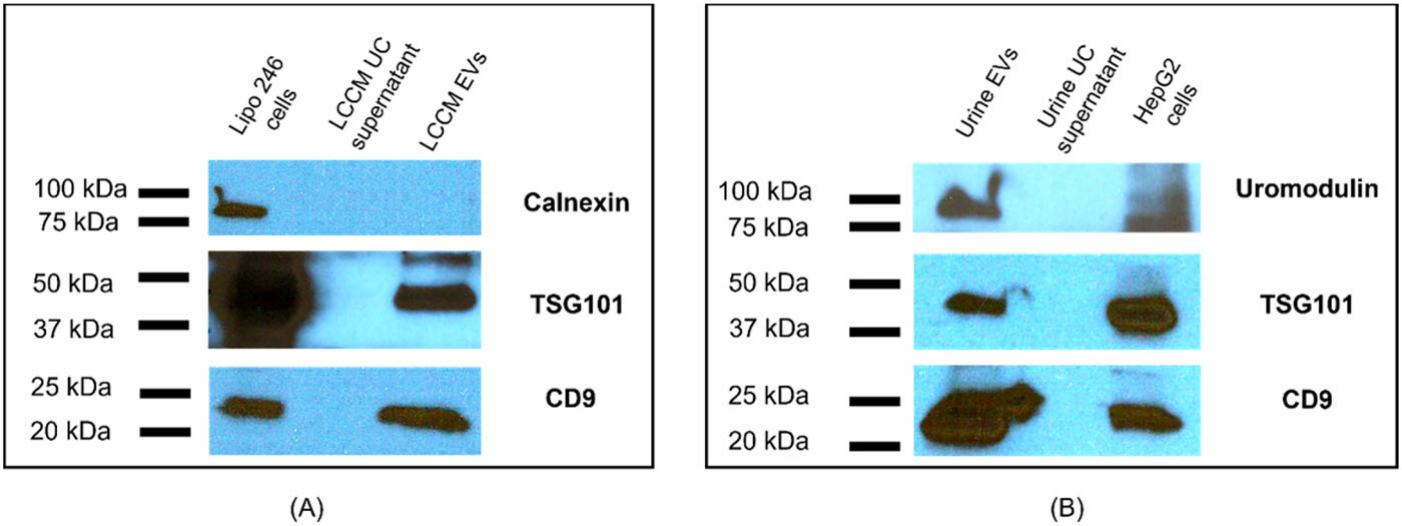
Western blot analysis of EV‐associated and control proteins in (A) Lipo246 cells, LCCM UC supernatant, Lipo246‐derived EVs (LCCM‐EVs), and (B) urine‐derived EVs (uEVs), urine UC supernatant, and HepG2 cells (positive control for uromodulin). Equal amounts of total protein (4 µg) were loaded for all EV samples and UC‐supernatants, whereas 25 µg of total protein was loaded for cell lysates. LCCM‐EVs showed the presence of EV markers CD9 and TSG101, while the endoplasmic reticulum marker Calnexin was not detected, indicating minimal cell contamination. uEVs showed enrichment of CD9 and TSG101, as well as the presence of uromodulin, a commonly co‐isolated urinary protein. These results are consistent with the expected protein profiles of EV‐enriched proteins in both LCCM‐EVs and uEV preparations.

Western blot analysis showed enrichment of the tetraspanin CD9 and the exosomal marker TSG101 (Aksamitiene et al. 2025) in both LCCM‐EV and uEV fractions (Figure 3). The absence of the cellular marker Calnexin in LCCM‐EVs confirmed minimal cellular contamination, validating the isolation protocol. For uEVs, uromodulin, a commonly co‐isolated protein (Harding et al. 2024), was detected in the UC pellet and the positive control Hep2G cells, consistent with expectations for uEV preparations.

### 3.2 Comparative Assessment of EVs

#### 3.2.1 LCCM‐EVs

All three instruments used for EV physical assessment detected an enrichment of small EVs (<200 nm) within isolated fractions, although the measured particle size and concentrations varied across instruments (Table 1). In scatter mode, total particle concentrations measured for all samples across instruments spanned two orders of magnitude, with ZetaView Evolution reporting the highest values, followed by CytoFLEX Nano and NanoFCM. For the unstained LCCM‐EVs, ZetaView Evolution reported the highest total particle concentration (4.59×10^9^ particles/mL), followed by the CytoFLEX Nano (2.67×10^8^ particles/mL) and NanoFCM (2.86×10^7^ particles/mL). When comparing the sizes reported by the three instruments, differences were again noted. The Cytoflex Nano reported a mean particle size of 77 ± 45 nm, while the NanoFCM reported 82 ± 22 nm, and the ZetaView Evolution reported 86 ± 54 nm. Median sizes (Table 1) followed a similar pattern.

**Table 1:** Summary table comparing LCCM‐EVs characteristics.

		Scatter mode			Fluorescence mode
Tetraspanin staining	Instrument	Total concentration (particles/mL)	Mean ± SD (nm)	Median (nm)	% population detected
None	CytoFLEX Nano	2.67 × 10^8^	77 ± 45	64	—
	NanoFCM	2.86 × 10^7^	82 ± 22	76	—
	ZetaView Evolution	4.59 × 10^9^	86 ± 54	71	—
CD81	CytoFLEX Nano	4.00 × 10^8^	106 ± 98	85	2.8
	NanoFCM	1.93 × 10^7^	75 ± 25	68	4.3
	ZetaView Evolution	5.52 × 10^9^	105 ± 82	88	3.3
CD63	CytoFLEX Nano	7.40 × 10^8^	96 ± 62	85	3.2
	NanoFCM	3.94 × 10^7^	91 ± 45	69	18.0
	ZetaView Evolution	2.16 × 10^9^	136 ± 115	113	9.8

In scatter mode, staining was associated with observed changes to both particle concentrations and size distributions. In LCCM‐derived EV samples, particle concentration increased following staining on the CytoFLEX Nano, whereas the NanoFCM and ZetaView Evolution exhibited non‐monotonic trends. In contrast, a consistent change in the measured particle sizes was observed. Across instruments, staining was associated with a shift toward larger mean and median particle diameters, particularly for CD63‐labeled samples (median ∼64–71 nm in unstained samples to ∼85–113 nm following staining), along with increased variability in size distributions.

Nevertheless, the overall concentration trend across instruments remained consistent, with ZetaView Evolution reporting the highest particle concentration, followed by the CytoFLEX Nano and NanoFCM, respectively. For CD81‐stained EV, mean particle sizes in scatter mode were 75 ± 25 nm (NanoFCM), 106 ± 98 nm (CytoFLEX Nano), and 105 ± 82 nm (ZetaView Evolution). The proportion of fluorescently positive CD81 EVs was highest for NanoFCM (4.3%), followed by ZetaView (3.3%) and CytoFLEX (2.8%). Similarly, for CD63‐stained EVs, mean particle sizes were 136 ± 115 nm (ZetaView Evolution), 96 ± 62 nm (CytoFLEX Nano), and 91 ± 45 nm (NanoFCM). The percentage of fluorescently positive CD63‐EVs also differed for each instrument, with the NanoFCM reporting fluorescent events at 18.0%, compared to ZetaView (9.8%) and CytoFLEX (3.2%). Antibody‐only process controls were run in parallel to account for any non‐specific signal from free antibodies and to define the fluorescent gating thresholds. Representative fluorescence scatter plots for CytoFLEX Nano and NanoFCM are shown in Figure S1 and S2, respectively.

#### 3.2.2 uEVs

Urine‐derived EVs from healthy donors were characterized in a manner similar to LCCM. Analysis of UC‐isolated urinary EVs as shown in Table 2 revealed greater heterogeneity in both particle size distribution and surface tetraspanin profile compared to LCCM‐derived EVs, reflecting the intrinsic compositional diversity of uEVs (Sedej et al. 2021). All three instruments detected broad EV populations spanning small (<200 nm) and larger (200–400 nm) vesicles. However, NanoFCM and CytoFLEX Nano predominantly detected EVs below 200 nm, with minimal contribution (<1%) from larger vesicles. In contrast, ZetaView Evolution captured a broader size range, with approximately ∼82% of particles <200 nm and ∼18% >200 nm.

In scatter mode, unstained uEVs showed the highest particle concentration for ZetaView Evolution (4.96×10^9^ particles/mL), followed by the CytoFLEX Nano (3.06×10^9^ particles/mL) and NanoFCM (9.59 × 10^8^ particles/mL). Mean particle sizes were 60 ± 40 nm (CytoFLEX Nano), 70 ± 19 nm (NanoFCM), and 165 ± 148 nm (ZetaView Evolution), with similar trends observed for the median particle sizes. Upon tetraspanin staining, rightward shifts in mean and median particle sizes were observed in scatter mode across all instruments, consistent with the addition of antibody complexes and labelling‐induced aggregation (Yim et al. 2023). For CD81‐stained EV preparations, NanoFCM measured the smallest mean size (67 ± 19 nm) and the lowest total concentration (4.96×10^8^ particles/mL) in scatter mode, whereas ZetaView Evolution reported larger mean sizes (123 ± 134 nm) and higher particle counts (2.75×10^9^ particles/mL). The proportion of CD81 EVs measured in fluorescent mode was highest for ZetaView (6.2%), followed by NanoFCM (0.4%) and CytoFLEX Nano (0.3%).

For CD63‐stained EVs, NanoFCM detected a mean size of 64 ± 14 nm and 1.8% positive events, while ZetaView Evolution showed a pronounced size shift (130 ± 121 nm) with 15.2% CD63‐positive EVs, and CytoFLEX Nano yielded intermediate size values (73 ± 38 nm) with a low fraction of positive events (0.5%). Representative fluorescence scatter plots for stained EVs and antibodies‐only controls are shown in Figures S1 and S2. Collectively, these findings highlight the instrument‐dependent variability in EV physical characteristics for both biofluids.

### 3.3 EV‐Associated Total DNA Quantification

#### 3.3.1 LCCM‐EV‐DNA

EV‐DNA was recorded using Nanodrop and Qubit. Each reported value represents the EV‐DNA measurement expressed as the mean ± standard deviation from three independent samples, with each sample analyzed on both instruments. Nanodrop quantification of EV‐DNA, normalized to the input volume of LCCM, yielded 14.10 ± 7.18 ng/mL. For the Nanodrop measurement, the corresponding purity ratio (A260/280) was 1.79 ± 0.12, values consistent with prior studies reporting acceptable purity for DNA samples (∼1.7–2.0) (Bruijns et al. 2022). Qubit‐based quantification of EV‐associated DNA yielded 0.12 ± 0.04 ng/mL.

#### 3.3.2 uEV‐DNA

uEVs were analyzed as EVs isolated from a complex, physiologically relevant biological fluid (Hinzman et al. 2022). To date, no universally accepted standard protocol exists for urine processing and subsequent EV isolation (Erdbrügger et al. 2021, Vall‐Palomar et al. 2018). The urine processing and EV isolation protocol described here follows previously published methodologies (Van Deun et al. 2017). Based on the results observed for LCCM‐EVs, uEV‐associated DNA was quantified from healthy donor samples (*n* = 4; labelled H1‐H4) following lysis at room temperature (RT). The total uEV‐DNA extracted from the UC pellets (Table 3) ranged from 0.2 to 0.7 ng/mL of urine. As in previous reports (Sedej et al., 2022 Sep 23, Blijdorp et al. 2021) EV‐associated DNA was normalized to urinary creatinine concentrations (Table 3).

## 4 Discussion

A major challenge facing the EV‐community is achieving standardized and reproducible characterization across different analytical instruments for respective biological EV‐sources (Hendrix et al. 2023, Ramirez et al. 2018). The growing diversity of instruments and isolation methods has advanced the ability to study EVs but has also introduced substantial variability in reported measurements of EV size, concentration, and molecular composition for the same samples, making it challenging to interpret results for similar pathologies and biological systems.

The three platforms used in this study (NanoFCM, CytoFLEX Nano, and Particle Metrix ZetaView Evolution) are commercially available instruments extensively employed for EV characterization. These systems rely on distinct detection principles and differ substantially in optical designs and calibration strategies (Nolan and Duggan 2017). Despite these differences, all three instruments consistently detected small EV populations (< 200 nm) in both LCCM‐derived and urine‐derived EV preparations. However, measurements of absolute particle size, size distribution, and concentration on the same isolated EV sample varied across measurements, likely reflecting differences in their underlying optical detection and analytical principles rather than true biological discrepancies (Arab et al. 2021, Yim et al. 2023, Mladenović et al. 2025).

Among the three instruments evaluated, one instrument (Particle Metrix ZetaView Evolution) is a nanoparticle tracking analysis (NTA) system, and the other two are considered nano‐flow cytometers (the NanoFCM, CytoFLEX Nano). NTA determines particle size by tracking Brownian motion under dark‐field illumination to derive the hydrodynamic diameter using the Stokes‐Einstein equation (Einstein 1906). In this system, scattered light is used solely for particle visualization, and measured sizes are influenced by particle diffusion, which is in turn affected by temperature and fluid and particle properties, such as viscosity (Filipe et al. 2010).

On the other hand, the nano‐flow cytometry (nFCM) platforms were developed from conventional cell‐based flow cytometry technologies (Orfao and Ruiz‐Argüelles 1996), and the intensity of scattered light is used to infer the particle hydrodynamic size (Rayleigh 1871). The light scattering signal depends on multiple parameters, including the illumination wavelength and power, collection angle and numerical aperture of the detector optics, quantum efficiency of the photodetector, and the refractive indices (RI) of both the particle and the surrounding medium (Welsh et al. 2023). Calibration is performed using reference beads with known size and concentration; thus, the accuracy of scatter‐derived EV diameters depends on the calibration translation to real EV suspensions.

Even with extensive calibration, the use of fundamentally distinct physical models without *a priori* knowledge of exact EV properties likely confounds estimates of particle size and concentration. In addition, unlike calibration beads, which are homogeneous and well‐defined, EVs are biologically complex particles characterized by heterogeneous membrane composition, surface proteins, and potential biomolecular corona (Tóth et al. 2021). These features can influence particle measurements in a technique‐dependent manner. In NTA‐based measurements, the Brownian motion‐derived hydrodynamic diameter includes contributions from surface‐associated molecules and the corona, which have been shown to attenuate EV motility in suspensions, and potentially lead to larger apparent sizes (Skliar et al. 2018). In contrast, flow cytometry‐based approaches estimate particle size indirectly from light‐scattering intensity, which may not fully account for surface‐associated features. Consistent with previous reports, ZetaView Evolution reported larger particle sizes than the nano‐flow cytometers for both LCCM and EVs and uEVs, as shown in Tables 1 and 2.

**Table 2:** Summary table comparing uEVs characteristics.

		Scatter mode			Fluorescence mode
Tetraspanin staining	Instrument	Total concentration (particles/mL)	Mean ± SD (nm)	Median (nm)	% population detected
None	CytoFLEX Nano	3.06 × 10^9^	60 ± 40	52	—
	NanoFCM	9.59 × 10^8^	70 ± 19	64	—
	ZetaView Evolution	4.96 × 10^9^	165 ± 148	127	—
CD81	CytoFLEX Nano	2.92 × 10^9^	66 ± 43	54	0.3
	NanoFCM	4.96 × 10^8^	67 ± 19	62	0.4
	ZetaView Evolution	2.75 × 10^9^	123 ± 134	91	6.2
CD63	CytoFLEX Nano	2.58 × 10^9^	73 ± 38	62	0.5
	NanoFCM	4.43 × 10^8^	64 ± 14	60	1.8
	ZetaView Evolution	1.97 × 10^8^	130 ± 121	107	15.2

**Table 3:** uEV‐associated DNA quantification.

Sample	U‐creatinine (mmol/L)	DNA/mL urine (ng/mL)	DNA/ U‐creatinine (ng/mmol)
H1	0.37	0.364 ± 0.009	982.801 ± 0.009
H2	0.35	0.697 ± 0.003	1991.342 ± 0.002
H3	0.40	0.195 ± 0.006	488.636 ± 0.013
H4	0.39	0.208 ± 0.003	532.246 ± 0.006

Previous inter‐platform comparison studies provide important context for the reported results in this work. Vogel et al. (Vogel et al. 2021) evaluated six orthogonal techniques using synthetic nanoparticles and plasma‐derived EVs and reported substantial variability in measured particle concentrations, with NTA yielding the highest apparent concentrations and NanoFCM among the lowest, and an overall coefficient of variation of ∼47% across platforms. Similarly, George et al. (George et al. 2021) observed that total events detected by NTA exceeded flow‐cytometric EV counts by one to two orders of magnitude, while Van der Pol et al. (van der Pol et al. 2014) reported approximately 15‐fold lower counts using conventional flow cytometry compared to NTA. Notably, conventional flow cytometers are constrained by size‐dependent detection limits and thus detect only a subset of the EV populations, leading to underestimation of vesicle concentrations due to limited sensitivity to smaller EVs (Nolan and Duggan 2018, van der Pol et al. 2018). In contrast, the higher apparent concentrations measured by NTA can be attributed to the diffusion‐based detection principle, which captures a broader range of particles, including smaller EVs and co‐isolated nanoscale components (Gupta et al. 2018, Seo et al. 2023). Scatter‐based detection in nanoflow cytometry, by comparison, is constrained by signal‐intensity thresholds (Zucker et al. 2016, Gao et al. 2022), leading to more selective but lower particle counts.

Furthermore, differences in the effective detection limits of each instrument can contribute to the observed variation in EV size and concentration measurements. NTA‐based systems (ZetaView Evolution), which rely on diffusion‐based tracking, can potentially detect smaller particles, in principle, to ∼10 nm; whereas nano‐flow cytometry platforms (NanoFCM and CytoFLEX Nano) rely on scatter‐based detection and typically detect particles down to ∼40 nm under optimized conditions. Operationally, these limits are dependent on instrument settings and are commonly defined using polystyrene standards; therefore, may not directly translate to EVs. The results reported in this work show that a fraction of particles (∼5%, ∼5 × 10^7^ particles/mL) falls below 40 nm in the NTA measurements, whereas both nano‐flow cytometry platforms predominantly detected particles above 40 nm. Consequently, while sub‐40 nm particles may contribute to the higher apparent concentrations measured by NTA, differences in detection sensitivity within the 40–150 nm size range, where the majority of EVs are distributed, are likely the primary drivers of the observed discrepancies across techniques.

Importantly, our results further show that variability persists even between nano‐flow cytometry platforms, highlighting the influence of instrument‐specific optical configurations and detection sensitivity. When comparing the two nano‐flow cytometers, NanoFCM consistently reported lower total particle concentrations and smaller mean sizes than the CytoFLEX Nano. The NanoFCM employs a 488 nm (10 mW) excitation laser and collects side‐scattered light with a single‐photon counting module (SPCM) filtered through a 488 nm bandpass (NanoFCM). In contrast, the CytoFLEX Nano uses a higher‐power 488 nm laser (50 mW) and dual side‐scatter detectors (VSSC1 and VSSC2), which collect scattered light through violet (405 nm‐centered) bandpass filters (Beckman‐Coulter). The inclusion of the violet‐side scatter channels enhances sensitivity to small vesicles by capturing shorter‐wavelength scattering, which exhibits higher relative intensity due to the strong inverse wavelength dependence of Rayleigh scattering (George et al. 2021). While the higher laser power in the CytoFLEX Nano improves the signal‐to‐noise ratio for weakly scattering particles, it may also elevate the apparent event rate and background, especially in heterogeneous EV samples (Welsh et al. 2023).

TEM provides image‐based reference measurements for EV size and enables direct visualization of vesicle morphology (Singh et al. 2024). Across both LCCM‐EVs and uEVs, the mean and median sizes reported by all three platforms were consistently larger than those obtained by TEM. Specifically, TEM measurements showed EV size ranging from 30–195 nm for LCCM‐EVs and 27–181 nm for uEVs, whereas the lowest reliably detectable particle sizes on the three instruments are above ∼40 nm due to instrument constraints (Arab et al. 2021). Consequently, TEM may allow visualization of smaller vesicle populations that are not accessible to the scatter‐ and tracking‐based platforms (Varga et al. 2014). However, TEM does not provide statistically robust size distributions and is subject to preparation‐induced artifacts, including selective adsorption, drying effects, and potential vesicle deformation (Rikkert et al. 2019). Therefore, TEM measurements are interpreted here as qualitative rather than quantitative descriptor of EV size or integrity.

Collectively, this study provides a direct, head‐to‐head comparison of multiple advanced EV characterization platforms, several of which have not previously been evaluated in the same work, demonstrating that differences in EV measurements arise from a combination of instrument design, operation, and the inherent biological complexity of EVs. The results reported in this work establish a systematic framework for interpreting and relating EV measurements across techniques.

Building on the existing literature, we evaluated detection events for two commonly used EV surface markers, CD63 and CD81 (Tables 1 and 2). For LCCM‐EVs, CD81 expression levels measured by CytoFLEX Nano and ZetaView Evolution were comparable to each other but lower than those reported by NanoFCM. In contrast, CD63 detection followed a different pattern, with CytoFLEX Nano reporting the lowest levels, followed by ZetaView Evolution, and NanoFCM detecting the highest levels. For uEVs, the trends changed again, with CD81 expression detected by Cytoflex Nano and NanoFCM relatively low, whereas ZetaView Evolution showed a much higher detection level. However, when CD63 expression levels were evaluated, the values obtained using CytoFlex Nano were lower than those reported by NanoFCM, while ZetaView Evolution yielded higher counts.

The measured fractions of CD63^+^ and CD81^+^ events in this study are at the lower end of reported ranges. However, the percentage of tetraspanin‐positive events was defined as the ratio of fluorescence‐positive events to the total number of particles detected in scatter mode, likely leading to an underestimation of CD63^+^ and CD81^+^ events (Woud et al. 2022). Notably, past reports with similar expression levels for CD63^+^ and CD81^+^ EVs (e.g., Dong et al. reported ∼1% CD81^+^ and ∼2% CD63^+^ uEV populations using NanoFCM following UC‐based isolation and post‐staining washing (Dong et al. 2020), while Yim et al. reported ∼2.2% CD81^+^ and ∼5% CD63^+^ populations (Yim et al. 2023)) have also been reported. The lower expression levels appear to be related to specific biofluid processing protocols (Takov et al. 2017, Yadav et al. 2026). Notably, despite lower absolute percentages of each tetraspannin type, the results here consistently show a relatively higher fraction of CD63^+^ relative to CD81^+^ EVs, also in agreement with prior reports (Yim et al. 2023, Anfaiha‐Sanchez et al. 2024).

The higher particle concentrations observed in stained samples compared to unstained samples suggest significant nuances for the measured quantities due to the underlying detection modality and sample composition. In scatter‐based measurements, the detected particle population is not restricted to fluorescently labeled EVs but includes all particles exceeding a pre‐defined scatter threshold, comprising both labeled and unlabelled EVs as well as co‐isolated non‐vesicular components such as protein aggregates, lipoproteins, and other components (Welsh et al. 2023, Sódar et al. 2016, van der Pol et al. 2018). Notably, no specific trend in particle concentrations across instruments was observed, suggesting that these differences are driven primarily by instrument‐specific detection characteristics and sample‐staining‐dependent factors. In contrast, the mean and median particle sizes increased following antibody labelling. The shift towards a larger measured size may be attributed to the formation of antibody‐associated complexes (Yim et al. 2023, Xu et al. 2024). Such complexes may arise from interactions between antibodies and EV surfaces or with co‐isolated components present in UC‐derived samples, leading to an increase in the effective hydrodynamic or apparent particle size (Lannigan and Erdbruegger 2017, Dlugolecka and Czystowska‐Kuzmicz 2024, Johnson et al. 2020). Notably, the absence of a comparable size shift in uEVs likely reflects differences arising from pre‐processing fluids used for EV isolation. Specifically, for uEVs, the sucrose cushion step has been reported previously to reduce co‐isolated components while also limiting non‐specific antibody interactions (Gupta et al. 2018, Seo et al. 2023, Teixeira‐Marques et al. 2024, Duong et al. 2019).

A critical factor contributing to the elevated fluorescent event counts detected with ZetaView Evolution in uEVs is the susceptibility of fluorescence‐based NTA to non‐specific background signals, as reported previously (Mladenović et al. 2025). Unlike nano‐flow cytometry, which records fluorescence only from particles passing through a focused interrogation point, fluorescence NTA detects all fluorescent objects within the imaging field (Mladenović et al. 2025). Consequently, any fluorescently labeled non‐vesicular material, including free dye, antibody‐dye complexes, lipoproteins, and protein aggregates, may be recorded as positive events (Yim et al. 2023).

One limitation of this study is the absence of a harmonized, cross‐instrument calibration using identical and shared reference samples. Although platform‐specific calibration procedures as recommended by manufacturers and prior studies were implemented, the lack of standardized calibration across instruments may contribute to differences in interpreting results across instruments. In addition, antibody‐only controls in buffer were used to assess baseline aggregation, but they do not account for potential interactions between antibodies and co‐isolated components in UC‐derived samples. Such interactions may contribute to background signals and influence both scatter‐ and fluorescence‐based measurements, particularly in complex biofluids.

Finally, EV‐DNA yields were also evaluated. Nanodrop measurements consistently overestimated the EV‐DNA levels relative to Qubit measurements, in agreement with prior comparative studies (Simbolo et al. 2013, Olson and Morrow 2012). This overestimation arises from the intrinsic limitations of absorbance‐based quantification, as absorbance at 260 nm does not distinguish between nucleotides, single‐ and double‐stranded DNA, RNA, or other UV‐absorbing contaminants (Gallagher 1998). Moreover, past reports have shown higher reliability for Qubit‐based DNA quantification compared to Nanodrop measurements (Versmessen et al. 2024, Masago et al. 2021). For a pilot analysis on uEVs derived from healthy donor urine samples, substantial variability in EV‐DNA amount was observed, consistent with previous reports suggesting that EV cargo distribution is heterogeneous across vesicles from individuals (Yokoi et al. 2019).

## 5 Summary and Conclusions

The field of EV‐research continues to expand rapidly, alongside a growing need for robust and reproducible characterization methods. Standardized approaches are essential to enable meaningful comparisons across studies and to facilitate the translation of emerging EV technologies into a variety of diagnostic and therapeutic applications. Results reported here, together with previous reports, emphasize that EV characterization is method‐dependent. Accurate interpretation, therefore, requires careful consideration of each platform's limitations and capabilities, as well as validation of the reported metrics using orthogonal methods.

## Disclosure

The information or content and conclusions do not necessarily represent the official position or policy of, nor should any official endorsement be inferred on the part of, The Uniformed Services University, the Department of War, The Ohio State University, or the US. Government.

## Conflicts of Interest

The authors declare no conflicts of interest.

## Supporting information




**Supporting Information Figure S1**: Representative CytoFLEX Nano fluorescence gating strategy for the detection of tetraspanin‐positive EVs. (A‐D) LPS246 cell line‐conditioned media EVs (LCCM‐EVs): (A) Y595 channel negative control, (B) B531 channel negative control, (C) CD81‐labeled EVs, and (D) CD63‐labeled EVs. (E‐H) Urine‐derived EVs (uEVs): (E) Y595 channel negative control, (F) B531 channel negative control, (G) CD81‐labeled uEVs, and (H) CD63‐labeled uEVs. (I‐J) PBS buffer‐only and (K‐L) antibody‐only controls were used to define background fluorescence and gating thresholds. Axes represent violet side scatter (VSSC1‐A) versus fluorescence intensity (Y595‐A or B531‐A). Percentages indicate the fraction of gated events relative to the total number of detected particles. **Supporting Information Figure S2**: Representative NanoFCM fluorescence gating strategy for the detection of tetraspanin‐positive EVs. (A‐D) LPS246 cell line‐ conditioned media EVs (LCCM‐EVs): (A) PE channel negative control, (B) FITC channel negative control, (C) CD81‐PE‐labeled EVs, and (D) CD63‐AF488‐labeled EVs (that is triggered in the FITC channel). (E‐H) Urine‐derived EVs (uEVs): (E) PE channel negative control, (F) FITC channel negative control, (G) CD81‐PE‐labeled uEVs, and (H) CD63‐AF488‐labelled EVs. (I‐J) PBS buffer‐only and (K‐L) antibody‐only controls were used to define background fluorescence and establish gating thresholds. Axes represent side scatter (SSC‐A) versus fluorescence intensity (PE‐A or FITC‐A). Percentages indicate the fraction of gated events relative to the total number of detected particles.

## Data Availability

The data that support the findings of this study are available from the corresponding author upon request.

## References

[jex270154-bib-0001] Adamczyk, A. M. , M. í. L. Leicaj , M. P. Fabiano , et al. 2023. “Extracellular Vesicles From Human Plasma Dampen Inflammation and Promote Tissue Repair Functions in Macrophages.” Journal of extracellular vesicles 12, no. 6: 12331. 37272889 10.1002/jev2.12331 PMC10241174

[jex270154-bib-0002] Aksamitiene, E. , J. Park , M. Marjanovic , and S. A. Boppart . 2025. “Defining Biological Variability, Analytical Precision and Quantitative Biophysiochemical Characterization of Human Urinary Extracellular Vesicles.” Journal of Extracellular Vesicles 14, no. 5: e70087. 40384173 10.1002/jev2.70087 PMC12086329

[jex270154-bib-0003] Anfaiha‐Sanchez, M. , A. Santiago‐Hernandez , J. A. Lopez , et al. 2024. “Urinary Extracellular Vesicles as a Monitoring Tool for Renal Damage in Patients Not Meeting Criteria for Chronic Kidney Disease.” Journal of Extracellular Biology 3, no. 9: e170. 39290459 10.1002/jex2.170 PMC11406310

[jex270154-bib-0004] Arab, T. , E. R. Mallick , Y. Huang , et al. 2021. “Characterization of Extracellular Vesicles and Synthetic Nanoparticles With Four Orthogonal Single‐Particle Analysis Platforms.” Journal of Extracellular Vesicles 10, no. 6: e12079. 33850608 10.1002/jev2.12079 PMC8023330

[jex270154-bib-0005] Arya, S. B. , S. P. Collie , and C. A. Parent . 2024. “The Ins‐and‐outs of Exosome Biogenesis, Secretion, and Internalization.” Trends in Cell Biology 34, no. 2: 90–108. 37507251 10.1016/j.tcb.2023.06.006 PMC10811273

[jex270154-bib-0006] Barreiro, K. , O. M. P. Dwivedi , G. Leparc , et al. 2020. “Comparison of Urinary Extracellular Vesicle Isolation Methods for Transcriptomic Biomarker Research in Diabetic Kidney Disease.” Journal of Extracellular Vesicles 10, no. 2: e12038. 33437407 10.1002/jev2.12038 PMC7789228

[jex270154-bib-0007] Bebelman, M. P. , M. J. Smit , D. M. Pegtel , and S. R. Baglio . 2018. “Biogenesis and Function of Extracellular Vesicles in Cancer.” Pharmacology & therapeutics 188: 1–11. 29476772 10.1016/j.pharmthera.2018.02.013

[jex270154-bib-0008] Becker, M. W. , L. D. Peters , T. Myint , et al. 2023. “Immune Engineered Extracellular Vesicles to Modulate T Cell Activation in the Context of Type 1 Diabetes.” Science Advances 9, no. 22: eadg1082. 37267353 10.1126/sciadv.adg1082 PMC10765990

[jex270154-bib-0009] Beckman‐Coulter . CytoFLEX Nano Specification Sheet.

[jex270154-bib-0010] Blijdorp, C. J. , O. A. Z. Tutakhel , T. A. Hartjes , et al. 2021. “Comparing Approaches to Normalize, Quantify, and Characterize Urinary Extracellular Vesicles.” Journal of the American Society of Nephrology 32, no. 5: 1210–1226. 33782168 10.1681/ASN.2020081142 PMC8259679

[jex270154-bib-0011] Bruijns, B. , T. Hoekema , L. Oomens , R. Tiggelaar , and H. Gardeniers . 2022. “Performance of Spectrophotometric and Fluorometric DNA Quantification Methods.” Analytica 3, no. 3: 371–384.

[jex270154-bib-0012] Buzas, E. I. 2023. “The Roles of Extracellular Vesicles in the Immune System.” Nature Reviews Immunology 23, no. 4: 236–250. 10.1038/s41577-022-00763-8 PMC9361922 35927511

[jex270154-bib-0013] Casadei, L. , F. Calore , D. A. Braggio , et al. 2019. “MDM2 Derived From Dedifferentiated Liposarcoma Extracellular Vesicles Induces MMP2 Production From Preadipocytes.” Cancer Research 79, no. 19: 4911–4922. 31387924 10.1158/0008-5472.CAN-19-0203 PMC6774856

[jex270154-bib-0014] Casadei, L. , F. Calore , C. J. Creighton , et al. 2017. “Exosome‐Derived miR‐25‐3p and miR‐92a‐3p Stimulate Liposarcoma Progression.” Cancer Research 77, no. 14: 3846–3856. 28588009 10.1158/0008-5472.CAN-16-2984 PMC6033276

[jex270154-bib-0015] Casadei, L. , A. Choudhury , P. Sarchet , et al. 2021. “Cross‐Flow Microfiltration for Isolation, Selective Capture and Release of Liposarcoma Extracellular Vesicles.” Journal of Extracellular Vesicles 10, no. 4: e12062. 33643547 10.1002/jev2.12062 PMC7887429

[jex270154-bib-0016] Casadei, L. , P. Sarchet , F. C. C. de Faria , et al. 2022. “In Situ Hybridization to Detect DNA Amplification in Extracellular Vesicles.” Journal of Extracellular Vesicles 11, no. 9: e12251. 36043432 10.1002/jev2.12251 PMC9428764

[jex270154-bib-0017] Crescitelli, R. , Y. Huang , A. N. Hendrix , et al. 2025. “Recommendations for Studying in Situ Extracellular Vesicles From Solid Tissue.” Journal of Extracellular Vesicles 14, no. 11: e70185. 41220186 10.1002/jev2.70185 PMC12606037

[jex270154-bib-0018] de Voogt, W. S. , R. Frunt , R. M. Leandro , et al. 2024. “EV‐Elute: A Universal Platform for the Enrichment of Functional Surface Marker‐Defined Extracellular Vesicle Subpopulations.” Journal of Extracellular Vesicles 13, no. 12: e70017. 39692115 10.1002/jev2.70017 PMC11653091

[jex270154-bib-0019] Dlugolecka, M. , and M. Czystowska‐Kuzmicz . 2024. “Factors to Consider Before Choosing EV Labeling Method for Fluorescence‐Based Techniques.” Frontiers in Bioengineering and Biotechnology 12: 1479516. 39359260 10.3389/fbioe.2024.1479516 PMC11445045

[jex270154-bib-0020] Dong, L. , R. C. Zieren , K. Horie , et al. 2020. “Comprehensive Evaluation of Methods for Small Extracellular Vesicles Separation From human Plasma, Urine and Cell Culture Medium.” Journal of Extracellular Vesicles 10, no. 2: e12044. 33489012 10.1002/jev2.12044 PMC7810129

[jex270154-bib-0021] Duong, P. , A. Chung , L. Bouchareychas , and R. L. Raffai . 2019. “Cushioned‐Density Gradient Ultracentrifugation (C‐DGUC) Improves the Isolation Efficiency of Extracellular Vesicles.” PLoS ONE 14, no. 4: e0215324. 30973950 10.1371/journal.pone.0215324 PMC6459479

[jex270154-bib-0022] Einstein, A. 1906. “On the Theory of the Brownian Movement.” Annalen der Physik 19, no. 4: 371–381.

[jex270154-bib-0023] Elzanowska, J. , L. Berrocal , B. García‐Peláez , et al. 2022. “Defining Optimal Conditions for Tumor Extracellular Vesicle DNA Extraction for Mutation Profiling.” Cancers 14, no. 13: 3258. 35805031 10.3390/cancers14133258 PMC9265681

[jex270154-bib-0024] Erdbrügger, U. , C. J. Blijdorp , I. V. Bijnsdorp , et al. 2021. “Urinary Extracellular Vesicles: A Position Paper by the Urine Task Force of the International Society for Extracellular Vesicles.” Journal of Extracellular Vesicles 10, no. 7: e12093. 34035881 10.1002/jev2.12093 PMC8138533

[jex270154-bib-0025] Filipe, V. , A. Hawe , and W. Jiskoot . 2010. “Critical Evaluation of Nanoparticle Tracking Analysis (NTA) by NanoSight for the Measurement of Nanoparticles and Protein Aggregates.” Pharmaceutical Research 27, no. 5: 796–810. 20204471 10.1007/s11095-010-0073-2 PMC2852530

[jex270154-bib-0026] Gallagher, S. 1998. “Quantitation of Nucleic Acids With Absorption Spectroscopy.” Current Protocols in Protein Science 13, no. 1: A.4K.1–A.4K.3. 10.1002/0471140864.psa04ks13 18429085

[jex270154-bib-0027] Gao, K. , H. Lian , C. Xue , J. Zhou , and X. Yan . 2022. “High‐Throughput Counting and Sizing of Therapeutic Protein Aggregates in the Nanometer Size Range by Nano‐Flow Cytometry.” Analytical Chemistry 94, no. 50: 17634–17644. 36474427 10.1021/acs.analchem.2c04382

[jex270154-bib-0028] Gassmann, H. , K. Schneider , V. Evdokimova , et al. 2021. “Ewing Sarcoma‐Derived Extracellular Vesicles Impair Dendritic Cell Maturation and Function.” Cells 10, no. 8: 2081. 34440851 10.3390/cells10082081 PMC8391167

[jex270154-bib-0029] George, S. K. , L. Lauková , R. Weiss , et al. 2021. “Comparative Analysis of Platelet‐Derived Extracellular Vesicles Using Flow Cytometry and Nanoparticle Tracking Analysis.” International Journal of Molecular Sciences 22, no. 8: 3839. 33917210 10.3390/ijms22083839 PMC8068037

[jex270154-bib-0030] Gheinani, A. H. , M. Vögeli , U. Baumgartner , et al. 2018. “Improved Isolation Strategies to Increase the Yield and Purity of Human Urinary Exosomes for Biomarker Discovery.” Scientific Reports 8, no. 1: 3945. 29500443 10.1038/s41598-018-22142-x PMC5834546

[jex270154-bib-0031] Gupta, S. , S. Rawat , V. Arora , et al. 2018. “An Improvised One‐Step Sucrose Cushion Ultracentrifugation Method for Exosome Isolation From Culture Supernatants of Mesenchymal Stem Cells.” Stem Cell Research & Therapy 9, no. 1: 180. 29973270 10.1186/s13287-018-0923-0 PMC6033286

[jex270154-bib-0032] Harding, M. A. , H. Yavuz , A. Gathmann , S. Upson , A. Swiatecka‐Urban , and U. Erdbrügger . 2024. “Uromodulin and the Study of Urinary Extracellular Vesicles.” Journal of Extracellular Biology 3, no. 11: e70022. 39582686 10.1002/jex2.70022 PMC11583080

[jex270154-bib-0033] Hendrix, A. N. , L. Lippens , C. L. Pinheiro , et al. 2023. “Extracellular Vesicle Analysis.” Nature Reviews Methods Primers 3, no. 1: 56. 10.1038/s43586-023-00240-z PMC12968065 41809514

[jex270154-bib-0034] Hinzman, C. P. , M. Jayatilake , S. Bansal , et al. 2022. “An Optimized Method for the Isolation of Urinary Extracellular Vesicles for Molecular Phenotyping: Detection of Biomarkers for Radiation Exposure.” Journal of Translational Medicine 20, no. 1: 199. 35538547 10.1186/s12967-022-03414-7 PMC9092707

[jex270154-bib-0035] Jiang, Y. , L. I. Yan , B. Zhou , et al. 2025. “Identifying Plasma Exosome Antigens as a Potential Diagnostic Biomarker for Tuberculosis Disease.” BMC Infectious Diseases 25, no. 1: 65. 39815174 10.1186/s12879-025-10474-9 PMC11734573

[jex270154-bib-0036] Johnson, S. M. , A. Banyard , C. Smith , A. Mironov , and M. G. McCabe . 2020. “Large Extracellular Vesicles Can be Characterised by Multiplex Labelling Using Imaging Flow Cytometry.” International Journal of Molecular Sciences 21, no. 22: 8723. 33218198 10.3390/ijms21228723 PMC7699300

[jex270154-bib-0037] Kalluri, R. , and V. S. LeBleu . 2020. “The Biology, Function, and Biomedical Applications of Exosomes.” Science 367, no. 6478: eaau6977. 32029601 10.1126/science.aau6977 PMC7717626

[jex270154-bib-0038] Kalluri, R. , and K. M. McAndrews . 2023. “The Role of Extracellular Vesicles in Cancer.” Cell 186, no. 8: 1610–1626. 37059067 10.1016/j.cell.2023.03.010 PMC10484374

[jex270154-bib-0039] Lang, J. B. , M. è C. Buck , J. Rivière , et al. 2022. “Comparative Analysis of Extracellular Vesicle Isolation Methods From human AML Bone Marrow Cells and AML Cell Lines.” Frontiers in Oncology 12: 949261. 36263223 10.3389/fonc.2022.949261 PMC9574064

[jex270154-bib-0040] Lannigan, J. , and U. Erdbruegger . 2017. “Imaging Flow Cytometry for the Characterization of Extracellular Vesicles.” Methods 112: 55–67. 27721015 10.1016/j.ymeth.2016.09.018

[jex270154-bib-0041] Lee, Y. J. , K. J. Shin , and Y. C. Chae . 2024. “Regulation of Cargo Selection in Exosome Biogenesis and Its Biomedical Applications in Cancer.” Experimental & Molecular Medicine 56, no. 4: 877–889. 38580812 10.1038/s12276-024-01209-y PMC11059157

[jex270154-bib-0042] Masago, K. , S. Fujita , Y. Oya , et al. 2021. “Comparison Between Fluorimetry (Qubit) and Spectrophotometry (NanoDrop) in the Quantification of DNA and RNA Extracted From Frozen and FFPE Tissues From Lung Cancer Patients: A Real‐World Use of Genomic Tests.” Medicina 57, no. 12: 1375. 34946321 10.3390/medicina57121375 PMC8709233

[jex270154-bib-0043] Mladenović, D. , J. Brealey , B. Peacock , K. Koort , and N. š. Zarovni . 2025. “Quantitative Fluorescent Nanoparticle Tracking Analysis and Nano‐Flow Cytometry Enable Advanced Characterization of Single Extracellular Vesicles.” Journal of Extracellular Biology 4, no. 1: e70031. 39790179 10.1002/jex2.70031 PMC11707551

[jex270154-bib-0044] Moon, S. , D. Shin , S. Kim , et al. 2019. “Enrichment of Exosome‐Like Extracellular Vesicles From Plasma Suitable for Clinical Vesicular miRNA Biomarker Research.” Journal of Clinical Medicine 8, no. 11: 1995. 31731761 10.3390/jcm8111995 PMC6912341

[jex270154-bib-0045] Motori, E. , I. Atanassov , S. M. V. Kochan , et al. 2020. “Neuronal Metabolic Rewiring Promotes Resilience to Neurodegeneration Caused by Mitochondrial Dysfunction.” Science advances 6, no. 35: eaba8271. 32923630 10.1126/sciadv.aba8271 PMC7455195

[jex270154-bib-0046] Musante, L. , S. V. Bontha , S. La Salvia , et al. 2020. “Rigorous Characterization of Urinary Extracellular Vesicles (uEVs) in the Low Centrifugation Pellet—A Neglected Source for uEVs.” Scientific Reports 10, no. 1: 3701. 32111925 10.1038/s41598-020-60619-w PMC7048852

[jex270154-bib-0047] NanoFCM . NanoAnalyzer Specification Sheet.

[jex270154-bib-0048] Nolan, J. P. , and E. Duggan . 2017. “Analysis of Individual Extracellular Vesicles by Flow Cytometry.” Flow cytometry protocols 1678: 79–92. 10.1007/978-1-4939-7346-0_5 29071676

[jex270154-bib-0049] Nolan, J. P. , and E. Duggan . 2018. “Analysis of Individual Extracellular Vesicles by Flow Cytometry.” in Flow Cytometry Protocols, edited by T.S. Hawley and R.G. Hawley , 79–92. Springer New York. 10.1007/978-1-4939-7346-0_5 29071676

[jex270154-bib-0050] Olson, N. D. , and J. B. Morrow . 2012. “DNA Extract Characterization Process for Microbial Detection Methods Development and Validation.” BMC Research Notes 5, no. 1: 668. 23206278 10.1186/1756-0500-5-668 PMC3599793

[jex270154-bib-0051] Orfao, A. , and A. Ruiz‐Argüelles . 1996. “General Concepts About Cell Sorting Techniques.” Clinical Biochemistry 29, no. 1: 5–9. 8929817 10.1016/0009-9120(95)02017-9

[jex270154-bib-0052] Palviainen, M. , M. Saraswat , Z. á. Varga , et al. 2020. “Extracellular Vesicles From human Plasma and Serum are Carriers of Extravesicular Cargo—Implications for Biomarker Discovery.” PLoS ONE 15, no. 8: e0236439. 32813744 10.1371/journal.pone.0236439 PMC7446890

[jex270154-bib-0053] Particle‐Metrix . Zetaview Evolution Specification Sheet.

[jex270154-bib-0054] Peng, T. , P. Zhang , J. Liu , et al. 2011. “An Experimental Model for the Study of Well‐Differentiated and Dedifferentiated Liposarcoma; Deregulation of Targetable Tyrosine Kinase Receptors.” Laboratory Investigation 91, no. 3: 392–403. 21060307 10.1038/labinvest.2010.185 PMC3058694

[jex270154-bib-0055] Qiagen . QIAamp DNA Micro Handbook. 2014.

[jex270154-bib-0056] Rädler, J. , D. Gupta , A. Zickler , and S. E. L. Andaloussi . 2023. “Exploiting the Biogenesis of Extracellular Vesicles for Bioengineering and Therapeutic Cargo Loading.” Molecular Therapy 31, no. 5: 1231–1250. 36805147 10.1016/j.ymthe.2023.02.013 PMC10188647

[jex270154-bib-0057] Ramirez, M. I. , M. G. Amorim , C. Gadelha , et al. 2018. “Technical Challenges of Working With Extracellular Vesicles.” Nanoscale 10, no. 3: 881–906. 29265147 10.1039/c7nr08360b

[jex270154-bib-0058] Rayleigh, L. 1871. “On the Light From the Sky, Its Polarization and Colour.” Phil Mag 41, no. 271: 107–120.

[jex270154-bib-0059] Rikkert, L. G. , R. Nieuwland , L. W. M. M. Terstappen , and F. A. W. Coumans . 2019. “Quality of Extracellular Vesicle Images by Transmission Electron Microscopy is Operator and Protocol Dependent.” Journal of Extracellular Vesicles 8, no. 1: 1555419. 30651939 10.1080/20013078.2018.1555419 PMC6327933

[jex270154-bib-0060] Santos, P. , and F. Almeida . 2021. “Exosome‐Based Vaccines: History, Current State, and Clinical Trials.” Frontiers in Immunology 12: 711565. 34335627 10.3389/fimmu.2021.711565 PMC8317489

[jex270154-bib-0061] Sedej, I. , M. Tušek Žnidarič , V. Dolžan , M. Lenassi , and M. Arnol . 2021. “Optimization of Isolation Protocol and Characterization of Urinary Extracellular Vesicles as Biomarkers of Kidney Allograft Injury.” Clinical Nephrology 96, no. 1: 107–113. 34643501 10.5414/CNP96S19

[jex270154-bib-0062] Sedej, I. , M. Štalekar , M. Tušek Žnidarič , et al. 2022. “Extracellular Vesicle‐Bound DNA in Urine is Indicative of Kidney Allograft Injury.” Journal of Extracellular Vesicles 11, no. 9: e12268. 36149031 10.1002/jev2.12268 PMC9503341

[jex270154-bib-0063] Seo, K. , J. I. H. Yoo , J. Kim , et al. 2023. “Ginseng‐Derived Exosome‐Like Nanovesicles Extracted by Sucrose Gradient Ultracentrifugation to Inhibit Osteoclast Differentiation.” Nanoscale 15, no. 12: 5798–5808. 36857681 10.1039/d2nr07018a

[jex270154-bib-0064] Shukla, S. , F. Currim , and R. Singh . 2023. “Do Different Exosome Biogenesis Pathways and Selective Cargo Enrichment Contribute to Exosomal Heterogeneity?” Biology of the Cell 115, no. 7: e2200116. 37179461 10.1111/boc.202200116

[jex270154-bib-0065] Simbolo, M. , M. Gottardi , V. Corbo , et al. 2013. “DNA Qualification Workflow for Next Generation Sequencing of Histopathological Samples.” PLoS ONE 8, no. 6: e62692. 23762227 10.1371/journal.pone.0062692 PMC3675123

[jex270154-bib-0066] Singh, P. K. , et al. 2025. “Electrokinetic‐Based Micro‐Nanofluidic Device for Sarcoma Extracellular Vesicle Isolation for Rapid Point‐of‐Care Detection.” 2025 23rd International Conference on Solid‐State Sensors, Actuators and Microsystems (Transducers).

[jex270154-bib-0067] Singh, P. K. , P. Sarchet , C. Hord , L. Casadei , R. Pollock , and S. Prakash . 2024. “Mechanical Property Estimation of Sarcoma‐Relevant Extracellular Vesicles Using Transmission Electron Microscopy.” Journal of Extracellular Biology 3, no. 7: e158. 38966868 10.1002/jex2.158 PMC11222873

[jex270154-bib-0068] Skliar, M. , V. S. Chernyshev , D. M. Belnap , et al. 2018. “Membrane Proteins Significantly Restrict Exosome Mobility.” Biochemical and Biophysical Research Communications 501, no. 4: 1055–1059. 29777705 10.1016/j.bbrc.2018.05.107

[jex270154-bib-0069] Sódar, B. W. , Á. Kittel , K. Pálóczi , et al. 2016. “Low‐Density Lipoprotein Mimics Blood Plasma‐Derived Exosomes and Microvesicles During Isolation and Detection.” Scientific Reports 6, no. 1: 24316. 27087061 10.1038/srep24316 PMC4834552

[jex270154-bib-0070] Takov, K. , D. M. Yellon , and S. M. Davidson . 2017. “Confounding Factors in Vesicle Uptake Studies Using Fluorescent Lipophilic Membrane Dyes.” Journal of extracellular vesicles 6, no. 1: 1388731. 29184625 10.1080/20013078.2017.1388731 PMC5699187

[jex270154-bib-0071] Teixeira‐Marques, A. , S. Monteiro‐Reis , D. Montezuma , et al. 2024. “Improved Recovery of Urinary Small Extracellular Vesicles by Differential Ultracentrifugation.” Scientific Reports 14, no. 1: 12267. 38806574 10.1038/s41598-024-62783-9 PMC11133306

[jex270154-bib-0072] Théry, C. , S. Amigorena , G. Raposo , and A. Clayton . 2006. “Isolation and Characterization of Exosomes From Cell Culture Supernatants and Biological Fluids.” *Current Protocols in Cell Biology* 30: Unit 3.22. 10.1002/0471143030.cb0322s30 18228490

[jex270154-bib-0073] Tóth, E. , L. Turiák , T. Visnovitz , et al. 2021. “Formation of a Protein Corona on the Surface of Extracellular Vesicles in Blood Plasma.” Journal of Extracellular Vesicles 10, no. 11: e12140. 34520123 10.1002/jev2.12140 PMC8439280

[jex270154-bib-0074] Tsering, T. , A. M. Nadeau , T. Wu , K. Dickinson , and J. V. Burnier . 2024. “Extracellular Vesicle‐Associated DNA: Ten Years Since Its Discovery in human Blood.” Cell Death & Disease 15, no. 9: 668. 39266560 10.1038/s41419-024-07003-y PMC11393322

[jex270154-bib-0075] Vall‐Palomar, M. , J. Arévalo , G. Ariceta , and A. Meseguer . 2018. “Establishment of Urinary Exosome‐Like Vesicles Isolation Protocol for FHHNC Patients and Evaluation of Different Exosomal RNA Extraction Methods.” Journal of Translational Medicine 16, no. 1: 278. 30305086 10.1186/s12967-018-1651-z PMC6180391

[jex270154-bib-0076] van der Pol, E. , F. A. Coumans , A. E. Grootemaat , et al. 2014. “Particle Size Distribution of Exosomes and Microvesicles Determined by Transmission Electron Microscopy, Flow Cytometry, Nanoparticle Tracking Analysis, and Resistive Pulse Sensing.” Journal of Thrombosis and Haemostasis 12, no. 7: 1182–1192. 24818656 10.1111/jth.12602

[jex270154-bib-0077] van der Pol, E. , L. de Rond , F. A. W Couman , et al. 2018. “Absolute Sizing and Label‐Free Identification of Extracellular Vesicles by Flow Cytometry.” Nanomedicine 14, no. 3: 801–810. 29307842 10.1016/j.nano.2017.12.012

[jex270154-bib-0078] van der Pol, E. , A. Sturk , T. van Leeuwen , et al. 2018. “Standardization of Extracellular Vesicle Measurements by Flow Cytometry Through Vesicle Diameter Approximation.” Journal of Thrombosis and Haemostasis 16, no. 6: 1236–1245. 29575716 10.1111/jth.14009

[jex270154-bib-0079] Van Deun, J. , P. Mestdagh , P. Agostinis , et al. 2017. “EV‐TRACK: Transparent Reporting and Centralizing Knowledge in Extracellular Vesicle Research.” Nature Methods 14, no. 3: 228–232. 28245209 10.1038/nmeth.4185

[jex270154-bib-0080] Van Niel, G. , G. d'Angelo , and G. Raposo . 2018. “Shedding Light on the Cell Biology of Extracellular Vesicles.” Nature reviews Molecular cell biology 19, no. 4: 213–228. 29339798 10.1038/nrm.2017.125

[jex270154-bib-0081] Varga, Z. , Y. Yuana , A. E. Grootemaat , et al. 2014. “Towards Traceable Size Determination of Extracellular Vesicles.” Journal of Extracellular Vesicles 3, no. 1: 23298. 10.3402/jev.v3.23298 PMC3916677 24511372

[jex270154-bib-0082] Versmessen, N. , L. Van Simaey , A. A. Negash , et al. 2024. “Comparison of DeNovix, NanoDrop and Qubit for DNA Quantification and Impurity Detection of Bacterial DNA Extracts.” PLoS ONE 19, no. 6: e0305650. 38885212 10.1371/journal.pone.0305650 PMC11182499

[jex270154-bib-0083] Vogel, R. , J. Savage , J. Muzard , et al. 2021. “Measuring Particle Concentration of Multimodal Synthetic Reference Materials and Extracellular Vesicles With Orthogonal Techniques: Who Is up to the Challenge?” Journal of Extracellular Vesicles 10, no. 3: e12052. 33473263 10.1002/jev2.12052 PMC7804049

[jex270154-bib-0084] Welsh, J. A. , G. J. A. Arkesteijn , M. Bremer , et al. 2023. “A Compendium of Single Extracellular Vesicle Flow Cytometry.” Journal of Extracellular Vesicles 12, no. 2: e12299. 36759917 10.1002/jev2.12299 PMC9911638

[jex270154-bib-0085] Welsh, J. A. , D. C. I. Goberdhan , L. O'Driscoll , et al. 2024. “Minimal Information for Studies of Extracellular Vesicles (MISEV2023): From Basic to Advanced Approaches.” Journal of Extracellular Vesicles 13, no. 2: e12404. 38326288 10.1002/jev2.12404 PMC10850029

[jex270154-bib-0086] Woud, W. W. , E. van der Pol , E. Mul , et al. 2022. “An Imaging Flow Cytometry‐Based Methodology for the Analysis of Single Extracellular Vesicles in Unprocessed Human Plasma.” Communications Biology 5, no. 1: 633. 35768629 10.1038/s42003-022-03569-5 PMC9243126

[jex270154-bib-0087] Xu, G. , J. Jin , Z. Fu , et al. 2025. “Extracellular Vesicle‐Based Drug Overview: Research Landscape, Quality Control and Nonclinical Evaluation Strategies.” Signal Transduction and Targeted Therapy 10, no. 1: 255. 40804047 10.1038/s41392-025-02312-w PMC12350758

[jex270154-bib-0088] Xu, S. , Z. Zhang , B. C. Melvin , N. Basu Ray , S. Ikezu , and T. Ikezu . 2024. “Comparison of Nanoimaging and Nanoflow Based Detection of Extracellular Vesicles at a Single Particle Resolution.” Journal of Extracellular Biology 3, no. 10: e70016. 39416671 10.1002/jex2.70016 PMC11481688

[jex270154-bib-0089] Yadav, A. , A. Sharma , M. Moulick , et al. 2026. “Labeling, Isolation and Characterization of Cell‐Type‐Specific Exosomes Derived From Mouse Skin Tissue.” Nature Protocols 21, no. 3: 1192–1234. 40940523 10.1038/s41596-025-01238-5 PMC12505222

[jex270154-bib-0090] Yáñez‐Mó, M. , P. R. Siljander , Z. Andreu , et al. 2015. “Biological Properties of Extracellular Vesicles and Their Physiological Functions.” Journal of Extracellular Vesicles 4, no. 1: 27066. 25979354 10.3402/jev.v4.27066 PMC4433489

[jex270154-bib-0091] Yim, K. H. O. W. , O. Krzyzaniak , A. Al Hrout , B. Peacock , and R. Chahwan . 2023. “Assessing Extracellular Vesicles in Human Biofluids Using Flow‐Based Analyzers.” Advanced Healthcare Materials 12, no. 32: e2301706. 37800440 10.1002/adhm.202301706 PMC11469288

[jex270154-bib-0092] Yokoi, A. , A. Villar‐Prados , P. A. Oliphint , et al. 2019. “Mechanisms of Nuclear Content Loading to Exosomes.” Science Advances 5, no. 11: eaax8849. 31799396 10.1126/sciadv.aax8849 PMC6867874

[jex270154-bib-0093] Zucker, R. M. , J. N. R. Ortenzio , and W. K. Boyes . 2016. “Characterization, Detection, and Counting of Metal Nanoparticles Using Flow Cytometry.” Cytometry Part A 89, no. 2: 169–183. 10.1002/cyto.a.22793 26619039

