# Supplementary material for: Evaluating Variability in Extracellular Vesicle Characterization Across Measurement Techniques
Source: J Extracell Biol. 2026 Jun 1;5(6):e70154. doi: 10.1002/jex2.70154 (PMC13239785; doi:10.1002/jex2.70154)
Supplement: Supplementary file 1 — Supporting Information Figure S1: Representative CytoFLEX Nano fluorescence gating strategy for the detection of tetraspanin‐positive EVs. (A‐D) LPS246 cell line‐conditioned media EVs (LCCM‐EVs): (A) Y595 channel negative control, (B) B531 channel negative control, (C) CD81‐labeled EVs, and (D) CD63‐labeled EVs. (E‐H) Urine‐derived EVs (uEVs): (E) Y595 channel negative control, (F) B531 channel negative control, (G) CD81‐labeled uEVs, and (H) CD63‐labeled uEVs. (I‐J) PBS buffer‐only and (K‐L) antibody‐only controls were used to define background fluorescence and gating thresholds. Axes represent violet side scatter (VSSC1‐A) versus fluorescence intensity (Y595‐A or B531‐A). Percentages indicate the fraction of gated events relative to the total number of detected particles. Supporting Information Figure S2: Representative NanoFCM fluorescence gating strategy for the detection of tetraspanin‐positive EVs. (A‐D) LPS246 cell line‐ conditioned media EVs (LCCM‐EVs): (A) PE channel negative control, (B) FITC channel negative control, (C) CD81‐PE‐labeled EVs, and (D) CD63‐AF488‐labeled EVs (that is triggered in the FITC channel). (E‐H) Urine‐derived EVs (uEVs): (E) PE channel negative control, (F) FITC channel negative control, (G) CD81‐PE‐labeled uEVs, and (H) CD63‐AF488‐labelled EVs. (I‐J) PBS buffer‐only and (K‐L) antibody‐only controls were used to define background fluorescence and establish gating thresholds. Axes represent side scatter (SSC‐A) versus fluorescence intensity (PE‐A or FITC‐A). Percentages indicate the fraction of gated events relative to the total number of detected particles. [file JEX2-5-e70154-s001.docx]

**Evaluating Variability in Extracellular Vesicle Characterization across Measurement Techniques**

Premanshu K. Singh^1, ¥^, Ali F. Usmani^1, ¥^, Debmalya Halder^1^, Jenna Miller^1^, Tharune Kanagasabai^1^, Patricia Sarchet^2^, Kevin Weller^2^, George J. Klarmann^3,4^, Raphael E. Pollock^2^, Federica Calore^2,5^, Shaurya Prakash^1, 2, †^

*^1^Department of Mechanical and Aerospace Engineering, The Ohio State University, Columbus, Ohio, USA; ^2^Comprehensive Cancer Center, The Ohio State University, Columbus, Ohio, USA; ^3^4DBio^3^ Center for Biotechnology, Department of Radiology and Bioengineering, Uniformed Services University of the Health Sciences, Bethesda, Maryland, USA; ^4^The Geneva Foundation, Tacoma, WA, USA; ^5^Department of Cancer Biology and Genetics, The James Comprehensive Cancer Center, The Ohio State University, Columbus, Ohio, USA.*

† Corresponding Author. E-mail: [prakash.31@osu.edu](mailto:prakash.31@osu.edu)

¥= Contributed equally

The figures presented next are referenced in the main manuscript and demonstrate the robustness of the results reported.


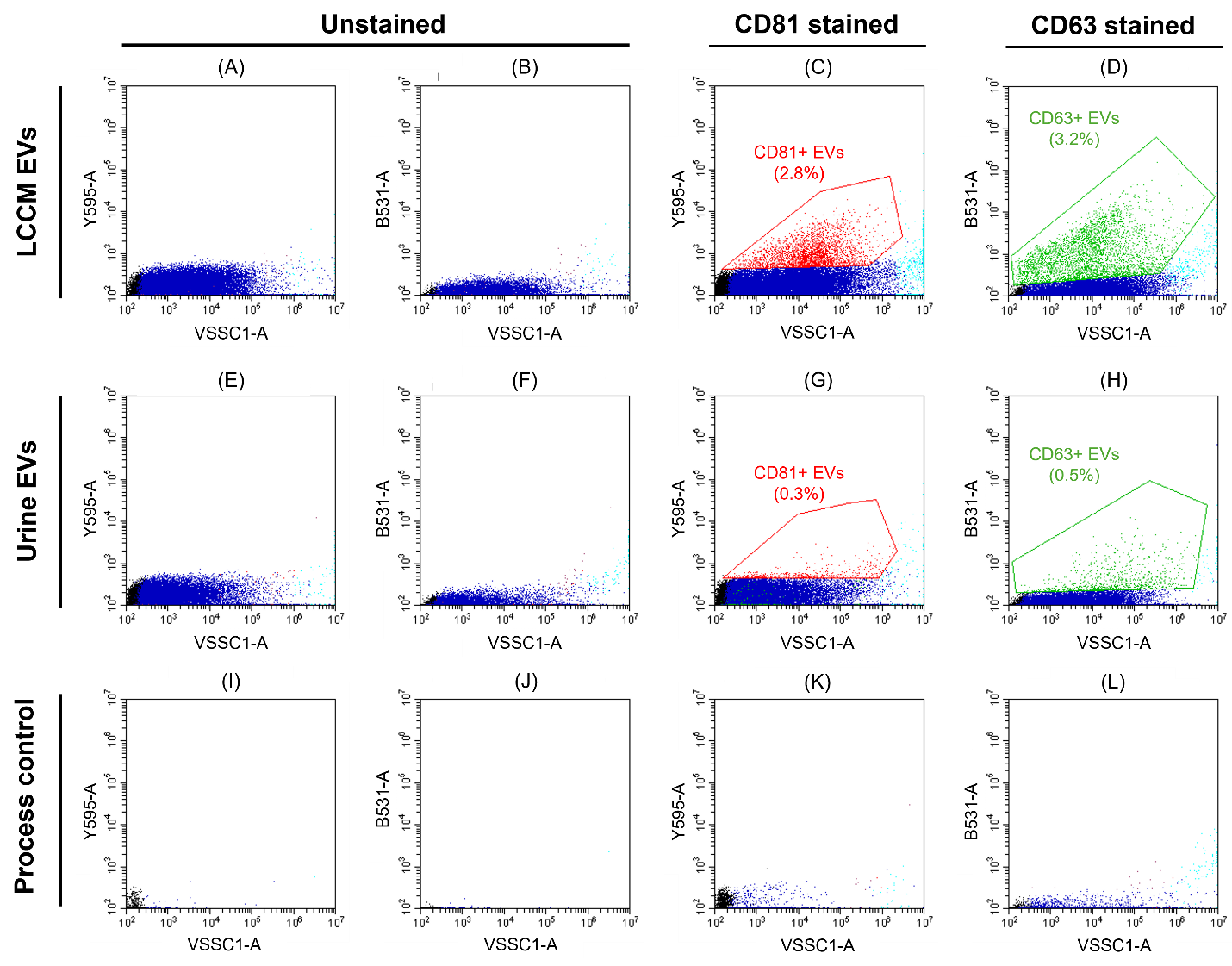
**Figure S1.** Representative CytoFLEX Nano fluorescence gating strategy for the detection of tetraspanin-positive EVs. (A-D) LPS246 cell line-conditioned media EVs (LCCM-EVs): (A) Y595 channel negative control, (B) B531 channel negative control, (C) CD81-labeled EVs, and (D) CD63-labeled EVs. (E-H) Urine-derived EVs (uEVs): (E) Y595 channel negative control, (F) B531 channel negative control, (G) CD81-labeled uEVs, and (H) CD63-labeled uEVs. (I-J) PBS buffer-only and (K-L) antibody-only controls were used to define background fluorescence and gating thresholds. Axes represent violet side scatter (VSSC1-A) versus fluorescence intensity (Y595-A or B531-A). Percentages indicate the fraction of gated events relative to the total number of detected particles.


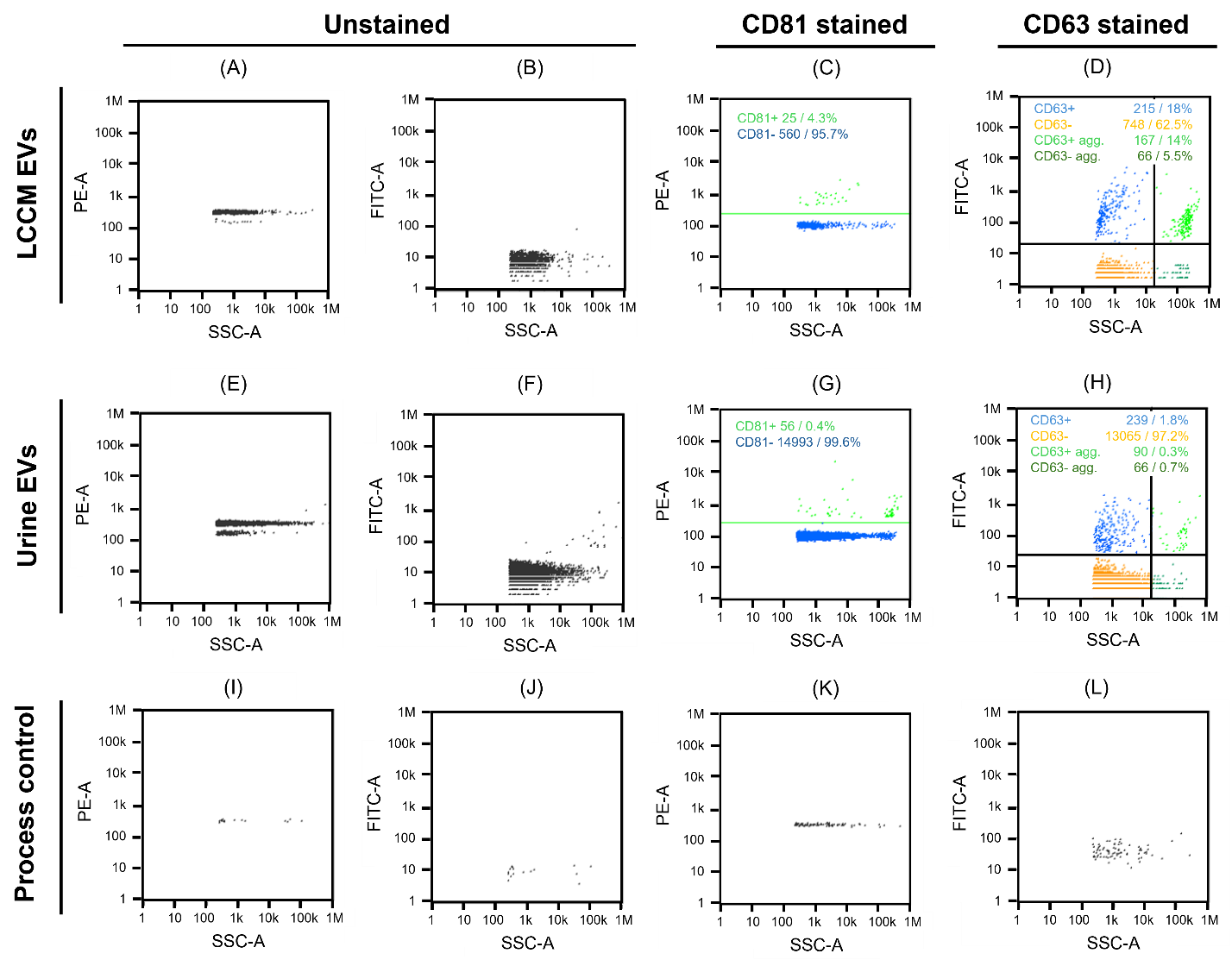


**Figure S2.** Representative NanoFCM fluorescence gating strategy for the detection of tetraspanin-positive EVs. (A-D) LPS246 cell line- conditioned media EVs (LCCM-EVs): (A) PE channel negative control, (B) FITC channel negative control, (C) CD81-PE-labeled EVs, and (D) CD63-AF488-labeled EVs (that is triggered in the FITC channel). (E-H) Urine-derived EVs (uEVs): (E) PE channel negative control, (F) FITC channel negative control, (G) CD81-PE-labeled uEVs, and (H) CD63-AF488-labelled EVs. (I-J) PBS buffer-only and (K-L) antibody-only controls were used to define background fluorescence and establish gating thresholds. Axes represent side scatter (SSC-A) versus fluorescence intensity (PE-A or FITC-A). Percentages indicate the fraction of gated events relative to the total number of detected particles.
